# Supplementary material for: All Set before Flowering: A 16S Gene Amplicon-Based Analysis of the Root Microbiome Recruited by Common Bean (Phaseolus vulgaris) in Its Centre of Domestication
Source: Plants (Basel). 2022 Jun 21;11(13):1631. doi: 10.3390/plants11131631 (PMC9269403; doi:10.3390/plants11131631)
Supplement: Supplementary file 1 [file plants-11-01631-s001.zip › plants-1538711-supplementary.pdf]

Supplementary material to Medina-Paz, F.; Herrera-Estrella, L.; Heil, M. All set before flowering: A 16S gene amplicon-based analysis of the root microbiome recruited by common bean (*Phaseolus vulgaris*) in its center of domestication. Plants 2022, 11, x. https://doi.org/10.3390/xxxxx

Tables S1, S2, S3, S6
Figure S1
Supplementary Methods 1

Table S1 Description of 16S rRNA gene amplicons of rhizosphere and endosphere samples of *Phaseolus vulgaris* subjected to sequencing on the MiSeq 2x300 Illumina platform

			**Description**				**Read numbers**	
**BioSample accession[1]**	**Code**	**Compartment**	**Stage**	**Replicate**	**Size**	**Raw**	**Reads 1**	**Reads 2**
SAMN23426372	VR1	Rhizosphere	Vegetative	1	774	1,010,079	933,482	812,089
SAMN23426373	VE1	Endosphere	Vegetative	1	803	986,255	946,019	869,969
SAMN23426374	FR1	Rhizosphere	Flowering	1	766	1,126,812	1,053,603	916,172
SAMN23426375	FE1	Endosphere	Flowering	1	858	881,729	836,106	768,793
SAMN23426376	PR1	Rhizosphere	Pod filling	1	788	1,013,548	944,181	799,497
SAMN23426377	PE1	Endosphere	Pod filling	1	894	794,281	749,457	688,024
SAMN23426378	VR2	Rhizosphere	Vegetative	2	777	1,005,419	941,504	807,296
SAMN23426379	VE2	Endosphere	Vegetative	2	827	966,592	929,127	853,062
SAMN23426380	FR2	Rhizosphere	Flowering	2	780	1,207,781	1,127,473	977,216
SAMN2342638	FE2	Endosphere	Flowering	2	846	991,475	940,805	852,042
**Sum**						**9,983,971**	**9,401,757**	**8,344,160**

[1]BioProject ID: PRJNA783305, Submission ID: SUB10705228

Table S2 BLAST annotations of sequences cloned from the ~750bp and the ~1300bp band of PCR products generated with primers 799f and 1492r from bean endosphere DNA

#	Clone	Acces-sion	Identity [%]	Length		MM	GO	Query		E	Sequence		Bit Score	Sequence Title
				Ali	Qu			start	end		Start	End		
**Sequences of clones from 750bp band**														
1	P15C5F	OL467029	95.80	731	762	1	0	21	751	0	66106	66836	1345.48	*Rhizobium etli* bv. *phaseoli* str. IE4803
	P15C5R	OL467030	95.80	731	762	1	0	12	742	0	66836	66106	1345.48	*Rhizobium etli* bv. *phaseoli* str. IE4803
2	P15C7F	OL467011	95.80	731	762	2	0	21	751	0	66106	66836	1339.94	*Rhizobium etli* bv. *phaseoli* str. IE4803
	P15C7R	OL467012	95.80	731	762	2	0	12	742	0	66836	66106	1339.94	*Rhizobium etli* bv. *phaseoli* str. IE4803
3	P15C10F	OL467013	95.80	731	762	2	0	21	751	0	66106	66836	1339.94	*Rhizobium etli* bv. *phaseoli* str. IE4803
	P15C10R	OL467014	95.80	731	762	2	0	12	742	0	66836	66106	1339.94	*Rhizobium etli* bv. *phaseoli* str. IE4803
4	P15C11F	OL467015	96.84	737	760	2	2	13	749	0	66102	66836	1343.63	*Rhizobium etli* bv. *phaseoli* str. IE4803
	P15C11R	OL467015	96.84	737	760	2	2	12	748	0	66836	66102	1343.63	*Rhizobium etli* bv. *phaseoli* str. IE4803
5	P15C12F	OL467017	95.41	731	762	10	1	24	751	0	66107	66836	1288.24	*Rhizobium etli* bv. *phaseoli* str. IE4803
	P15C12R	OL467018	95.92	731	760	8	1	11	740	0	66836	66106	1304.85	*Rhizobium etli* bv. *phaseoli* str. IE4803
6	P15C13F	OL467019	95.80	731	762	2	0	21	751	0	66106	66836	1339.94	*Rhizobium etli* bv. *phaseoli* str. IE4803
	P15C13R	OL467020	95.80	731	762	2	0	12	742	0	66836	66106	1339.94	*Rhizobium etli* bv. *phaseoli* str. IE4803
7	P16C1F	OL467021	96.84	737	760	2	2	13	749	0	66102	66836	1343.63	*Rhizobium etli* bv. *phaseoli* str. IE4803
	P16C1R	OL467022	96.84	737	760	2	2	12	748	0	66836	66102	1343.63	*Rhizobium etli* bv. *phaseoli* str. IE4803
8	P16C2F	OL467023	95.80	731	762	1	0	21	751	0	66106	66836	1345.48	*Rhizobium etli* bv. *phaseoli* str. IE4803
	P16C2R	OL467024	95.80	731	762	1	0	12	742	0	66836	66106	1345.48	*Rhizobium etli* bv. *phaseoli* str. IE4803
9	P16C3F	OL467025	95.21	399	418	13	3	20	418	0	1483	1088	645.60	*Rhizobium miluonense* str.
	P16C3R	OL467026	93.57	615	653	19	6	42	653	0	648	1259	1014.93	*Rhizobium* sp. strain MaGu46-1

10	P16C4F	OL467027	99.86	730	761	1	0	21	750	0	66836	66107	1343.63	*Rhizobium etli* bv. *phaseoli* str. IE4803
	P16C4R	OL467028	95.79	730	761	1	0	12	741	0	66107	66836	1343.63	*Rhizobium etli* bv. *phaseoli* str. IE4803
11	P16C5F	OL467029	99.86	731	762	0	0	21	751	0	66836	66107	1351.02	*Rhizobium etli* bv. *phaseoli* str. IE4803
	P16C5R	OL467030	99.86	731	762	0	0	21	751	0	66107	66836	1351.02	*Rhizobium etli* bv. *phaseoli* str. IE4803
12	P16C6F	OL467031	99.86	722	749	0	0	17	738	0	66115	66836	1334.40	*Rhizobium etli* bv. *phaseoli* str. IE4803
	P16C6R	OL467032	99.86	722	749	0	0	12	733	0	66836	66115	1334.40	*Rhizobium etli* bv. *phaseoli* str. IE4803
13	P16C7F	OL467033	97.46	731	762	2	0	21	751	0	66106	66836	1339.94	*Rhizobium etli* bv. *phaseoli* str. IE4803
	P16C7R	OL467034	97.46	731	762	2	0	12	742	0	66836	66106	1339.94	*Rhizobium etli* bv. *phaseoli* str. IE4803
**Samples from 1300bp band**														
1	P18C1F	OL468634	99.89	950	970	1	0	21	970	0	107461	106512	1749.90	*P. vulgaris* mitochondrion, compl. genome
	P18C1R	OL468635	99.91	1181	1212	2	0	21	1201	0	107461	106281	2170.93	*P. vulgaris* mitochondrion, compl. genome
2	P18C3F	OL468636	97.54	873	894	3	0	22	894	0	106281	107153	1596.63	*P. vulgaris* mitochondrion, compl. genome
	P18C3R	OL468637	97.28	1181	1213	4	0	22	1202	0	106281	107461	2159.85	*P. vulgaris* mitochondrion, compl. genome
3	P18C5F	OL468638	97.86	963	983	3	0	21	983	0	106281	107243	1762.82	*P. vulgaris* mitochondrion, compl. genome
	P18C5R	OL468639	97.36	1181	1212	3	0	21	1201	0	106281	107461	2165.39	*P. vulgaris* mitochondrion, compl. genome
4	P18C6F	OL468640	97.83	905	924	2	0	20	924	0	106281	107185	1661.26	*P. vulgaris* mitochondrion, compl. genome
	P18C6R	OL468641	90.64	1056	1164	9	0	20	1075	0	106281	107336	1901.32	*P. vulgaris* mitochondrion, compl. genome
5	P18C7F	OL468642	97.84	951	971	3	0	21	971	0	106281	107231	1740.66	*P. vulgaris* mitochondrion, compl. genome
	P18C7R	OL468643	97.44	1181	1211	2	0	21	1201	0	106281	107461	2165.39	*P. vulgaris* mitochondrion, compl. genome
6	P21C1F	OL468644	97.80	933	953	3	0	21	953	0	106281	107213	1707.42	*P. vulgaris* mitochondrion, compl. genome
	P21C1R	OL468645	98.77	962	973	2	0	12	973	0	107461	106500	1766.52	*P. vulgaris* mitochondrion, compl. genome
7	P21C2F	OL468646	97.83	904	923	3	0	20	923	0	106281	107184	1653.87	*P. vulgaris* mitochondrion, compl. genome
	P21C2R	OL468647	97.44	965	976	2	0	12	963	0	107461	106497	1772.06	*P. vulgaris* mitochondrion, compl. genome
8	P21C3F	OL468648	97.85	959	979	2	0	21	979	0	106281	107293	1760.98	*P. vulgaris* mitochondrion, compl. genome
	P21C3R	OL468649	98.80	981	992	1	0	12	992	0	107461	106481	1807.14	*P. vulgaris* mitochondrion, compl. genome
9	P21C4F	OL468650	97.70	895	915	3	0	21	915	0	106282	107176	1637.25	*P. vulgaris* mitochondrion, compl. genome
	P21C4R	OL468651	99.90	969	980	2	0	12	980	0	107461	106493	1779.44	*P. vulgaris* mitochondrion, compl. genome
10	P21C6F	OL468652	98.77	969	980	2	0	12	980	0	107461	106493	1779.44	*P. vulgaris* mitochondrion, compl. genome

	P21C6R	OL468653	98.80	991	1001	3	1	12	1001	0	107461	106471	1812.68	*P. vulgaris* mitochondrion, compl. genome
11	P21C7F	OL468654	97.67	882	902	2	0	21	902	0	106281	107162	1618.79	*P. vulgaris* mitochondrion, compl. genome
	P21C7R	OL468655	98.75	949	960	1	0	12	960	0	107461	106513	1748.05	*P. vulgaris* mitochondrion, compl. genome
12	P21C9F	OL468656	97.70	848	867	2	0	20	867	0	106281	107128	1556.00	*P. vulgaris* mitochondrion, compl. genome
	P21C9R	OL468657	99.70	922	924	1	0	3	924	0	107461	106540	1703.73	*P. vulgaris* mitochondrion, compl. genome
13	P22C3F	OL468658	99.89	968	988	3	0	21	988	0	106281	107248	1772.06	*P. vulgaris* mitochondrion, compl. genome
	P22C3R	OL468659	98.81	999	1010	2	0	12	1010	0	107461	106463	1834.84	*P. vulgaris* mitochondrion, compl. genome
14	P22C6F	OL468660	97.88	973	993	2	0	21	993	0	106281	107253	1786.83	*P. vulgaris* mitochondrion, compl. genome
	P22C6R	OL468661	98.75	954	965	2	0	12	965	0	107461	106508	1751.74	*P. vulgaris* mitochondrion, compl. genome
15	P22C7F	OL468662	97.73	906	926	2	0	21	926	0	106281	107186	1663.10	*P. vulgaris* mitochondrion, compl. genome
	P22C7R	OL468663	97.73	906	990	2	0	12	990	0	107461	106483	1797.91	*P. vulgaris* mitochondrion, compl. genome

*abbreviations: Al, alignment; E, E-value; GO, gap open; MM, mismatch; Qu, Query

Table S3. Diversity parameters of prokaryotic communities in rhizosphere and endosphere samples of *Phaseolus vulgaris*.

Sample ID	Name	Compartment	Stage	Replicate	Observed	H'	λ	J'
CA1MFSS01-01-S1_L001_R1_001	VE2	Endosphere	Vegetative	2	965	2.7858	0.90844	0.38262
CA1MFSS02-02-S1_L001_R1_001	VR2	Rhizosphere	Vegetative	2	3883	6.8972	0.96610	0.77237
CA1MFSS03-03-S1_L001_R1_001	FE2	Endosphere	Flowering	2	1017	2.7996	0.90849	0.38219
CA1MFSS04-04-S1_L001_R1_001	FR2	Rhizosphere	Flowering	2	4060	6.7600	0.99505	0.74773
CA1MFSS05-05-S1_L001_R1_001	PE1	Endosphere	Pod filling	1	493	2.9105	0.91670	0.43893
CA1MFSS06-06-S1_L001_R1_001	PR1	Rhizosphere	Pod filling	1	10703	7.1621	0.99631	0.77193
CA1MFSS07-07-S1_L001_R1_001	VE1	Endosphere	Vegetative	1	1403	3.0901	0.91979	0.39860
CA1MFSS08-08-S1_L001_R1_001	VR1	Rhizosphere	Vegetative	1	3853	6.9301	0.99632	0.77489
CA1MFSS09-09-S1_L001_R1_001	FE1	Endosphere	Flowering	1	1139	2.9977	0.92243	0.40329
CA1MFSS10-10-S1_L001_R1_001	FR1	Rhizosphere	Flowering	1	3916	6.9223	0.99650	0.77067

H', Shannon index; λ, Simpson's index; J', Evenness

Table S6. Sequences of degenerate primers used for PCR-based confirmation of predicted functional genes (Table 3)

Function	Gene	Primer sequenecs	PCR product	Annealing temp.	Ref.
Nitrogenase	nifH	F: 5' – ADN GCC ATC ATY TCN CC - 3'<br>R: 5' – TGY GAY CCN AAR GCN G - 3'	359bp	54°C	[140]
GlcNAc transferase	nodC	F: 5' - TG ATY GAY ATG GAR TAY TGG CT – 3'<br>R: 5' - CGY GAC ARC CAR TCG CTR TTG - 3'	640 bp	65°C-50°C	[141]
Chemotaxis and motility	motA	F: 5' - GTB CTS GGS GTS ATC AAG G - 3'<br>R: 5' - GGC GAR ARC AAG GCG GC - 3'	293 bp	54°C	This study
Butanediol dehydrogenase	butB	F: 5' - CAC GAT CAT GTG CGC GAA G - 3'<br>R: 5' - CGC GTG CAM TGG TGC GG - 3'	453 bp	46°C	This study
Chemotaxis and motility	gspD	R: 5' - CGC GTG CAM TGG TGC GG - 3'<br>F: 5 ' - ACG TGC TGT CGA CGC CGA A - 3'	427 bp	50°C	This study

Supplementary Figure S1.

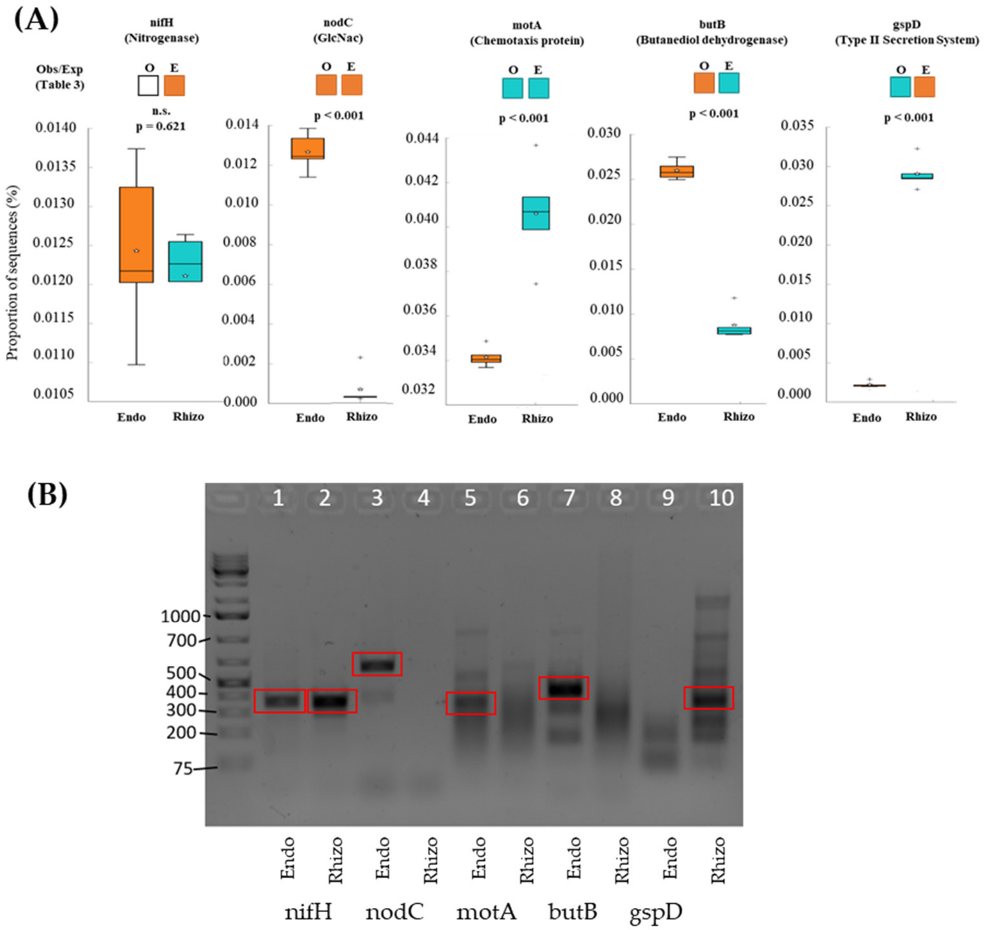

Supplementary Figure S1. Enrichment of predicted functional genes in the endosphere versus rhizosphere. (A) The proportion of each gene is expressed in percent (%) of all sequences in the inferred metagenome of each sample and presented as boxplots for the endosphere (orange) versus rhizosphere (blue). Insets repeat the observed (O) and expected (E) enrichment of each gene as presented in Table 3, p-values reports the results of Welch's t-test adjusted for multiple comparisons with the FDR correction using the Benjamin-Hochberg method (n = 5 biologically independent replicates per compartment).

(B) Agarose gel illustrating the amplification products obtained with degenerate primers (see Table S6), red squares highlight the band of the predicted size.

gspD, general secretion pathway protein D; motA, motility protein A; butB, butanediol dehydrogenase; nodC, nodulation gene C, nifH, nitrogen fixing H.

Supplementary Text S1. PCR conditions for the amplification of five key genes selected to validate predicted functions:
We used degenerate primers (see Table S6) to amplify each gene separately for each compartment, using as template pooled DNA prepared as equimolar mixture of the total DNA in the five original samples per compartment.

For nifH, we used the PCR protocol published for the respective primers [140]. The 25 µl PCR reaction mixture contained approximately 20 ng of total DNA for rhizosphere samples and the same amount for root endosphere samples, 10X PCR reaction buffer, $MgCl_2$ 2 mM, 1 µM of each primer, 100 µM each dNTP, and 1.5U of Taq Platinum DNA Polymerase (Invitrogen). After initial denaturation at 94°C for 15 min., each thermal cycle was performed as follows: denaturation at 94°C for 1 min., annealing at 54°C for 45 sec, and elongation 72°C for 1 min. At the end of 34 cycles, the final extension step was at 72°C for 10 min.

For nodC, we applied a "touchdown" PCR protocol following description by Sariota et al [141]. The 25 µl PCR reaction mixture contained approximately 10 ng of total DNA for rhizosphere samples and 10 ng for root endosphere samples, 10X PCR reaction buffer, $MgCl_2$ 2 mM, 1 µM of each primer, 100 µM each dNTP, and 1.5U of Taq Platinum DNA Polymerase (Invitrogen). Initial denaturation for 3 min at 94 °C, then 1 cycle of 96 °C for 20 sec, 65 °C for 30 sec and 72 °C for 30 sec, 2 cycles 96 °C for 20 sec, 62 °C for 30 sec and 72 °C for 35 sec, then 3 cycles of 96 °C for 20 sec, 59 °C for 30 sec and 72 °C for 40 sec, then 4 cycles of 96 °C for 20 sec, 56 °C for 30 sec and 72 °C for 45 sec, then 5 cycles 96 °C for 20 sec, 53 °C for 30 sec and 72 °C for 50 sec, then 25 cycles of 94 °C for 20 sec, 50 °C for 30 sec and 72 °C for 60 sec, and a final elongation at 72 °C for 5min.

For genes gspD, butB and motA, the 25 µl PCR reaction mixture contained approximately 10 ng of total DNA for rhizosphere samples and 10 ng for root endosphere samples, 10X PCR reaction buffer, $MgCl_2$ 2 mM, 1 µM of each primer, 100 µM each dNTP, and 1.5U of Taq Platinum DNA Polymerase (Invitrogen). After initial denaturation at 94°C for 3 min., each thermal cycle was performed as follows: denaturation at 94°C for 30 sec, annealing at temperatures shown at Table S6 for 45 sec, and elongation 72°C for 1 min. At the end of 34 cycles, the final extension step was at 72°C for 5 min.
